# Prenatal Exposure to Lipopolysaccharide Induces PTX3 Expression and Results in Obesity in Mouse Offspring

**DOI:** 10.1007/s10753-017-0626-1

**Published:** 2017-08-02

**Authors:** Shugang Qin, Xin Chen, Meng Gao, Jianzhi Zhou, Xiaohui Li

**Affiliations:** Institute of Materia Medical, College of Pharmacy, Third Military Medical University, Chongqing, 400038 China

**Keywords:** lipopolysaccharide, PTX3, obesity, Prenatal inflammation

## Abstract

This study tested the hypothesis whether inflammation will directly lead to obesity. This study was designed to investigate the relationship between inflammation and obesity by intraperitoneally injecting pregnant mice with lipopolysaccharide (LPS) (75 μg kg^−1^). The results showed that inflammation during pregnancy could lead to a significant increase in the levels of the inflammatory factor PTX3. The offspring of the LPS-treated mice displayed abnormal levels of fat development, blood lipids, and glucose metabolism, and fat differentiation markers were significantly increased. Our study also confirmed that PTX3 can increase the susceptibility to obesity by regulating the expression of adipogenic markers; this regulatory role of PTX3 is most likely caused by MAPK pathway hyperactivation. Our study is the first to find strong evidence of inflammation as a cause of obesity. We determined that PTX3 was an important moderator of obesity, and we elucidated its mechanism, thus providing new targets and theories for obesity therapy. Moreover, our study provides new ideas and directions for the early intervention of anti-inflammation in pregnancy.

## INTRODUCTION

As society develops, a series of cardiovascular and cerebrovascular diseases and metabolic diseases has been attributed to obesity, which has become an important factor that negatively affects human health [1]. The University of Washington Health Statistics Assessment study reported that the current global population of approximately 7 billion people have 2.1 billion people obese and that this is an increasing trend [2, 3]. Obesity is caused by a variety of factors and is a chronic metabolic disease that is characterized by increases in fat mass, visceral fat wet weight, fat coefficient, lipid metabolism disorders, and adipose cell volume and number, which lead to abnormal body weight and fat deposition in different parts of the body [4, 5]. An increasing number of studies have shown that obesity is a chronic inflammatory disease; when the excess fat cannot be metabolized, the body will mistakenly believe that the fat is external bacteria or other pathogens and immediately use the cellular immune system (mainly macrophages) to remove it, and therefore, adipose tissue is often associated with macrophage infiltration. During this process, increased synthesis of inflammatory factors leads to systemic inflammation and a variety of metabolic diseases [6–8].

Pentraxin-3 (PTX3) is the first long pentraxin protein that is induced by interleukin-1 (IL-1) in endothelial cells [9]. Tumor necrosis factor (TNF) can induce PTX3 expression in fibroblasts and adipose tissue [10, 11]. Anti-inflammatory cytokines IL-10 and high-density lipoprotein (HDL) can also induce cells to produce PTX3. This pentraxin is an essential component of the humoral arm of innate immunity, and it can be produced by a variety of cells in the inflammatory site, including dendritic cells, macrophages, fibroblasts, and activated endothelial cells [10–15]. Correlation analysis showed that lipopolysaccharide could induce PTX3 gene transcription in isolated mononuclear cells [16]. PTX3 activates and regulates the complement cascade by interacting with C1q and Factor H; therefore, it plays a non-redundant role in the resistance against selected microbes, cancer, tissue remodeling, and metabolic disease and in the regulation of inflammation [17–21]. Numerous studies have shown that PTX3 plays a different role in different species, tissues, metabolic pathways, and types of diseases and that it usually exhibits duality [22]. For example, in response to the influenza virus and giant cells, PTX3 is able to bind to IRF3 (interferon-3) by activating the expression of TLR9/MyD88 and IL-12 (interleukin-12), thereby enhancing the immune resistance of humans and mice to protect the body and reduce the risk of pathogens [23]. The latest research also shows that PTX3 deficiency triggers complement-dependent tumor-promoting inflammation, with enhanced tumor burden, macrophage infiltration, cytokine production, angiogenesis, and genetic instability, which increases tumor susceptibility [24]. However, in a model of *Klebsiella pneumoniae* infection in mice, PTX3 overexpression was reported to play antagonistic roles and increase lethality when the bacterial concentration was excessive [25].

The expression and balance of PTX3 play an important role in the occurrence and development of metabolic diseases [26]. Many studies have demonstrated that the prenatal environment during pregnancy is an independent factor affecting adult obesity [27–29]. Previous work from our group has also shown that a single injection of LPS inflammatory immune stimulation in pregnant mice results in hypertension, increased leptin levels, and increased body and fat tissue weight in the offspring [29–31]. However, the activity of PTX3 and the specific mechanisms in obese individuals remain unknown. Therefore, changes in the PTX3 gene and protein expression levels in the local adipose tissue were studied to explore the role and mechanism of the inflammation and obesity resulting from prenatal exposure to LPS.

## MATERIALS AND METHODS

### Animals

Nulliparous, time-mated C57 mice were purchased from the Animal Center of the Third Military Medical University (Chongqing, China) and were raised to the age of 8 weeks, when the weights of the females and males were 20 ± 2 and 25 ± 2 g, respectively, before mating one female to one male by placing them together in a cage overnight. The next day, the mice were separated, fed, and recorded, and the day was designated 0 days pregnant. The breeding mice were deemed pregnant following measurement of their body weight on the 11th day if their weight was significantly increased by greater than 2 g, and if an obvious increase in the size of the mouse abdomen could be observed. Pregnant mice were randomly divided into two groups (*n* = 10 in each): The NS group pregnant mice were injected intraperitoneally (Sigma Chemical, St. Louis, MO, USA) with 0.2 ml normal saline on the 11th day of gestation, and the LPS group pregnant mice were injected intraperitoneally with 75 μg kg^−1^ of LPS on the 11th day of pregnancy. The offspring mice were used as study subjects.

### Cell Culture

DMEM high-glucose medium (10%, HyClone, Logan, Utah, USA) was used to cultivate 3T3-L1 cells to 80% confluence. Induction reagent I was added to the culture medium for 48 h, and then induction reagent II was added to the culture medium for 48 h. Afterwards, the culture medium was replaced with normal medium, and the cells were cultured for 12 h before they were collected for real-time PCR and Western blot. Induction reagent I comprised 10% DMEM high glucose + 0.5 mmol l^−1^ isobutylmethylxanthine (IBMX) (Sigma Chemical, St. Louis, MO, USA) + 0.1 μmol l^−1^ dexamethasone (DEX) (Sigma Chemical, St. Louis, MO, USA) + 0.1 μmol l^−1^ insulin (INS) (Sigma Chemical, St. Louis, MO, USA); induction reagent II comprised 10% DMEM high glucose medium + 0.1 μmol l^−1^ INS.

### Body Weight

The body weight of the offspring mice was regularly monitored at 2-week intervals from 2 to 12 weeks of age during the experiments.

### Adipose Tissue Wet Weight, Coefficient, and Distribution

The 4-, 8-, and 12-week-old mouse offspring were euthanized by cervical dislocation after a final weight was obtained. The thoracic and abdominal cavities were cut, and the adipose tissue from the kidneys and subcutaneous and visceral fat of female mice and from the epididymal surface and subcutaneous and visceral fat of male mice was removed using tweezers. The tissue was washed with 0.9% saline and placed on filter paper for desiccation. Next, the wet weights of the female perirenal, subcutaneous, and visceral fat and male epididymis and subcutaneous and visceral adipose tissue were measured (g). The fat coefficient of adipose tissue was calculated as follows: fat coefficient = (wet weight of adipose tissue / body weight) × 100.

### HE Staining

The same mouse tissue samples from the 4-, 8-, and 12-week-old offspring mice were collected and incubated in 4% paraformaldehyde solution for 48 h before being dehydrated and embedded in paraffin wax. The tissue was sliced into 4-mm sections, and HE staining was performed. Morphological changes in adipose cells were observed under a light microscope, and the number and diameter (mm) of cells were recorded.

### Oral Glucose Tolerance Test

After fasting for 10 h, the 4-, 8-, and 12-week-old offspring mice were challenged with 2 g/kg of 50% glucose solution for the oral glucose tolerance test (OGTT). Blood samples were collected at 0, 30, 60, 120, and 180 min. Glucose levels were determined using an Accu-Chek glucose meter (Roche Diagnostics Ltd. Shanghai, China), and we calculated the area under the curve (AUC) for glucose.

### Determination of Lipid Levels

Blood was obtained from the retro-orbital plexus of the eye under anesthesia and prepared for hematological and biochemical examination. Serum total cholesterol (TC), triglyceride (TG), high-density lipoprotein (HDL-C), and low-density lipoprotein (LDL-C) levels were measured in the Laboratory Department of the Xinqiao Hospital (Chongqing, China).

### Real-Time PCR

The expression levels of the messenger RNA (mRNA) encoding adaptin 2 (AP2), peroxisome proliferator-activated receptor γ (PPARγ), CCAAT/enhancer-binding protein α (CEBP/α), CCAAT/enhancer-binding protein β (CEBP/β), and pentraxin-3 (PTX3) were assessed *via* real-time PCR when the mouse offspring were 4, 8, 12 weeks of age. Total RNA was extracted from the kidneys using an RNA simple Total RNA Kit (TIANGEN Biotech, Beijing, China) and then quantified by measuring the absorbance at 260 nm. Then, total RNA (1 μg) was reverse transcribed into cDNA using a PrimeScript™ RT Reagent Kit with gDNA Eraser (TaKaRa Biotechnology, Dalian, China). GAPDH was used as an internal control. The PCR primers were designed using Premier 5.0 (PREMIER Biosoft International, Palo Alto, CA, USA) and according to published nucleotide sequences. The sequences of the primers used in this study are presented in Table 1. Each real-time PCR reaction was conducted in a total volume of 25 μl containing SYBR® Premix Ex Taq™ II (Tli RNase H Plus) (TaKaRa Biotechnology, Dalian, China) in an Eppendorf Mastercycler ep realplex system (Eppendorf, Hamburg, Germany) under the following conditions: 30 s at 95 °C followed by 40 cycles of 95 °C for 15 s, 60 °C for 15 s, and 72 °C for 20 s. After amplification, melting curve analysis was performed by collecting fluorescence data while increasing the temperature from 65 to 99 °C over a period of 135 s. The relative expression ratio of each mRNA was calculated using the formula 1/2^ΔΔCt^.

**Table 1:** Primers Used in RT-PCR

Seq name	5′-3′ forward	5′-3′ reverse
CEBP/α	TCAAGGGCTTGGCTGGTCC	CGCGATGTTGTTGCGTTCC
CEBP/β	CACCGGGTTTCGGGACTTG	CCCGCAGGAACATCTTTAA
AP2	ATGAAAGAAGTGGGAGTTGGC	CAGTTTGAAGGAAATCTCGGT
PPARγ	ATGACAGACCTCAGGCAGATT	TGTCAGCGACTGGGACTTTTC
PTX3	TCTGTTCCTGAGGGTGGACT	CGACATTTCCCCGGATGTGA
siRNA negative control	UUCUCCGAACGUGUCACGUTT	ACGUGACACGUUCGGAGAATT
siRNA	GGAGCCCAGUAUGUUUCUUTT	AAGAAACAUACUGGGCUCCTT
Beta-actin	ACGGTCAGGTCATCACTATCG	GGCATAGAGGTCTTTACGGATG

### Western Blot

Total protein was extracted from the adipose tissue of 4-, 8-, and 12-week-old mouse offspring, and the protein concentrations were measured using the bicinchoninic acid (BCA) method. After denaturation and electrophoresis on sodium dodecyl sulfate (SDS)-polyacrylamide gels, the separated proteins were transferred to nitrocellulose membranes. Then, the membranes were blocked with 5% non-fat milk in TBST for 1 h. After incubation with the primary antibodies against AP2 (SC-12633; Santa Cruz, USA), PPARγ (sc-7196; Santa Cruz, USA), CEBP/α (sc-61; Santa Cruz, Dallas, Texas, USA), CEBP/β (sc-150; Santa Cruz, Dallas, Texas, USA), PTX3 (E5121-5B7, Abnova, New Brunswick, Canada), and GAPDH (Sigma Chemical, St. Louis, MO, USA) in TBS at 4 °C overnight, the membranes were incubated with a peroxidase-conjugated secondary antibody in TBS at room temperature for 1 h. Specific bands were detected *via* a chemiluminescence assay and recorded on X-ray film. Quantity One software (Bio-Rad, Philadelphia, PA, USA) was used to quantify the band intensities.

### Oil Red O Staining

At 48 h after transfection (cell numbers >10^5^), the DMEM was discarded, and the cells were collected and incubated in 4% paraformaldehyde solution for 30 min. After the paraformaldehyde was discarded, the cells were washed in 500 μl of water and then stained with 0.3% oil red O in isopropanol at 37 °C for 1 h. Cells were then washed with PBS. The cells were microscopically observed, and photographs were acquired. Then, 250 μl of isopropyl alcohol (IPA) was added to each well, after which 200 μl of the solution was transferred to a 96-well plate, and the OD value was measured at 500 nm. A stock solution of 0.5% oil red O and isopropanol was prepared by mixing 0.5 g of oil red O powder (Sigma Chemical, St. Louis, MO, USA) with 100 ml of isopropanol. The 0.3% oil red O isopropanol-water solution was prepared by mixing 12 ml of 0.5% oil red O and isopropanol stock solution with 8 ml of distilled water, followed by 0.22-μm microfiltration membrane filtration.

### Cell Apoptosis

The cells were collected 48 h after transfection (cell numbers >10^5^), washed twice with PBS, resuspended, and centrifuged 4 min at 3000 r. Then, 100 μl of fluorescent dye was added to each sample, and the cells were incubated at room temperature in the dark for 15 min and analyzed within 30 min. Fluorescent dye was prepared by mixing 100 μl of 1× binding buffer, 2 μl of Annexin V-FITC (BD Biosciences, San Diego, CA, USA), and 2 μl of propidium iodide (BD Biosciences, San Diego, CA, USA).

### Cell Differentiation

The cells were collected 48 h after transfection (cell numbers >10^5^), washed twice with PBS, and resuspended with 1 ml precooled 75% ethanol. The cells were fixed for 12 h overnight at 4 °C and then centrifuged for 5 min at 3000 r. The supernatant was discarded, and the cells were washed twice with PBS. Eighty microliters of 50 μg/μl RNase (BD Biosciences, San Diego, CA, USA) was added to each sample and incubated for 30 min at 37 °C. Then, 100 μl of propidium iodide (BD Biosciences, San Diego, CA, USA) was added to each sample and incubated at room temperature for 15 min. The propidium iodide signal was detected after 100-μm filter filtration.

### Cell Proliferation Was Measured by MTT Assay

The cells were collected 48 h after transfection (cell numbers >10^5^). After the DMEM was discarded, 100 μl of detection solution was added to each sample and incubated for 4 h at 37 °C in an incubator. Absorption of the supernatant was determined after adding 150 μl DMSO (China Chongqing East Chemical, Chongqing, China), followed by oscillation for 10 min. A microplate reader was used to determine the OD value at 490 nm. The detection solution consisted of 5 mg/ml MTT (Sigma Chemical, St. Louis, MO, USA), 100 mg of PMSF (Sigma Chemical, St. Louis, MO, USA), and 20 ml of PBS.

### Transfection with siRNA and PTX3 Overexpression Plasmid

The blank group was cultured with normal medium. The induced group was cultured with induction reagent I. The short interfering RNA (siRNA) control group was cultured with induction reagent I in Opti-MEM culture medium (1734724; Gibco, USA) containing Lipofectamine®3000 (1769228; Invitrogen, Shanghai, China) and NC (GenePharma, Shanghai, China). The siRNA group was cultured with induction reagent I in Opti-MEM culture medium containing Lipofectamine®3000 and siRNA (GenePharma, Shanghai, China). The PTX3 overexpression plasmid control group was cultured with induction reagent I in Opti-MEM culture medium containing Lipofectamine® 3000, PM3000 diluent, and the PTX3 empty vector (GenePharma, Shanghai, China). The PTX3 overexpression plasmid group was cultured with induction reagent I in Opti-MEM culture medium containing Lipofectamine®3000, P3000™ diluent (1769230; Invitrogen; Shanghai, China), and the PTX3 overexpression plasmid (GenePharma, Shanghai, China: NM_008987.3).

### Statistics

All of the data are expressed as the means ± SEM and were analyzed using the SPSS 13.0 software package (SPSS, Chicago, IL, USA). Comparisons between the groups were performed using one-way ANOVA followed by Fisher’s least significant difference (LSD) *post hoc* test. *p <* 0.05 was considered significant. *p <* 0.01 was considered significant.

## RESULTS

### The Effects of Maternal Inflammation During Pregnancy on the Fat Development and Distribution of Offspring Mice

**Fig. 1:**
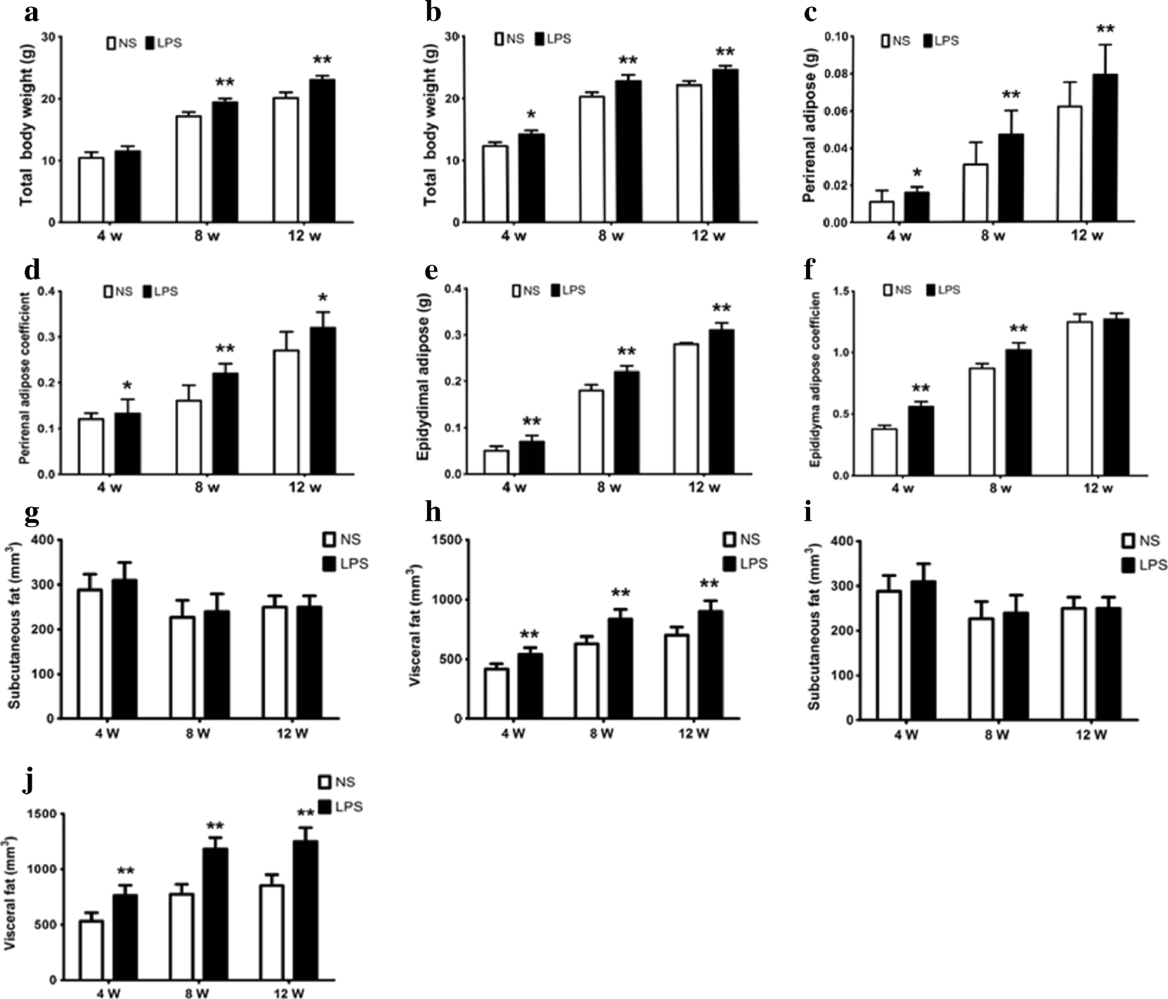
Characterization of maternal inflammation during pregnancy on the fat development and adipose distribution of offspring mice. Pregnant mice were administered intraperitoneally (i.p) with saline (NS group) or LPS (0.79 mg/kg, LPS group) at gestational 11 days. The body weight of the offspring mice was regularly monitored at 2-week intervals, the body weight of the offspring mice at different ages (**a** Weights of the female mice; **b** weights of the male mice). Perirenal fat wet weight (**c**) and fat coefficient (**d**) in the female offspring. Epidydimal fat wet weight (**e**) and fat coefficient (**f**) in the male offspring. All of the offspring mice subcutaneous fat and visceral fat wet weight. **g**, **h** Female mice. **i**, **j** Male mice).*p < 0.05, **p < 0.01, *compared with the control group (x ± s, n = 36).*

It is well known that obesity is associated with body weight and fat weight. In order to determine the effect of pregnant inflammation on mice fat development, we measured the offspring mice’ body weight, fat wet weight, and fat distribution. Compared with the control mice, the body weight was markedly increased in the LPS group in females at 8 and 12 weeks of age and males at 4, 8, and 12 weeks of age (*p <* 0.05) (Fig. 1a, b). All of the female offspring had significantly increased perirenal adipose tissue wet weight and fat coefficient of adipose tissue at 8 and 12 weeks in the LPS group compared with the NS group (*p <* 0.01) (Fig. 1c, d). All of the male offspring had significantly increased epididymis adipose tissue wet weight and fat coefficient of adipose tissue at 8 and 12 weeks of age in the LPS group compared with the NS group (*p <* 0.01) (Fig. 1e, f). LPS group offspring mouse visceral fat had significantly increased and had almost identical subcutaneous fat compared with the NS group (*p <* 0.05) (Fig. 1g–j).

### HE Staining Analysis of Adipocyte Morphology of Offspring Mice

**Fig. 2:**
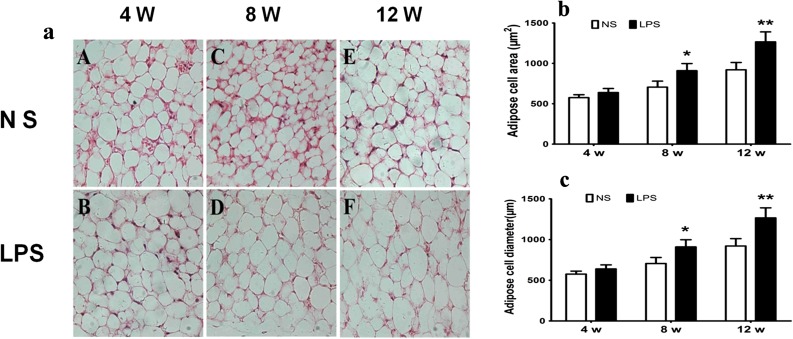
Prenatal exposure to LPS results in the change of adipocyte morphology in mouse offspring. Comparison of the fat cells in offspring mice (HE staining, ×40). Adipocyte morphology (cell diameter and area) was assessed by HE staining (×400) in the offspring fresh frozen section of male mice epidydimal fat. Morphological changes in adipose cells were observed under a light microscope, and the number and diameter (mm) of cells were recorded. The HE staining results are shown in **a**. (**a** Four-week-old NS group. **b** Four-week-old LPS group. **c** Eight-week-old NS group. **d** Eight-week-old LPS group. **e** Twelve-week-old NS group. **f** Twelve-week-old LPS group). Adipose cell diameter (**b**) and area (**c**) in the offspring mice.*p < 0.05, **p < 0.01*, compared with the control group (x ± s, n = 12).*

The volume of fat cells becomes larger which are the classic pathophysiological bases of obesity [31]. We then determined whether there is variation in the adipocyte morphology in the LPS group; the diameters and areas of adipose cells were significantly increased in the male mice at 4 and 8 weeks of age in the LPS group (*p <* 0.05) (Fig. 2a–c).

### The Effects of Prenatal Exposure to LPS on Lipid Levels of Offspring Mice

**Fig. 3:**
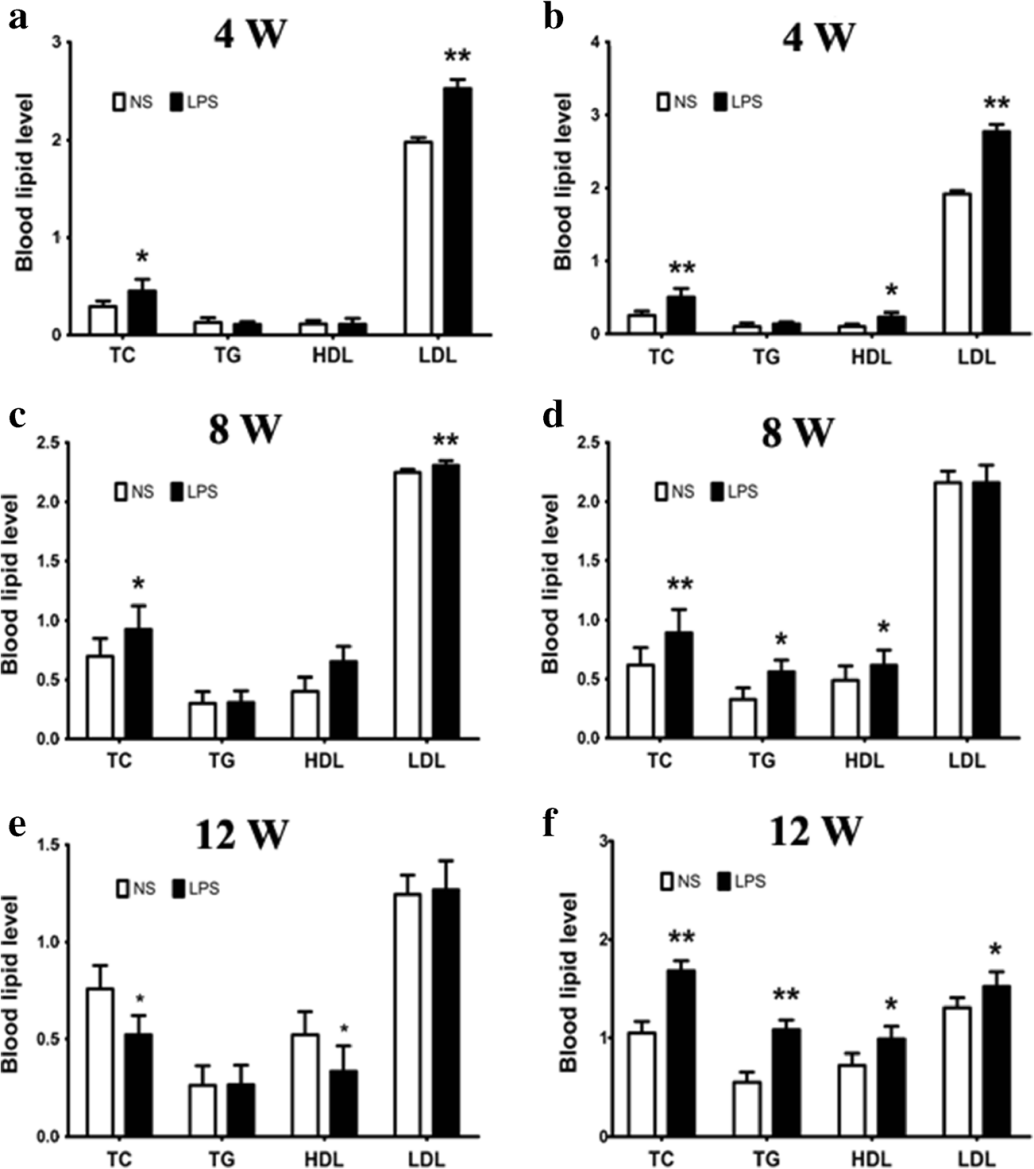
All of the offspring mice’ levels of blood lipid. The offspring female and male blood lipid levels were measured in the Laboratory Department of the Xinqiao Hospital (Chongqing, China). Blood lipid levels in 4-week-old offspring mice (**a** female mice; **b** male mice). Blood lipid levels of 8-week-old offspring mice (**c** female mice; **d** male mice). Blood lipid levels of 12-week-old offspring mice (**e** female mice; **f** male mice).*p < 0.05, **p < 0.01*, compared with the control group (x ± s, n = 16).*

It is well accepted that obesity will lead to lipid metabolism disorders and is involved in a variety of blood lipid metabolism [32]. The experiment result shows that the levels of TG and LDL in female mice at 4 and 8 weeks of age were significantly increased (*p <* 0.05) (Fig. 3a–e), and the levels of TC, TG, and LDL were significantly increased in male mice at every age tested (*p <* 0.05) (Fig. 3b–f).

### Prenatal Exposure to LPS Influence Glucose Metabolism of Offspring Mice

**Fig. 4:**
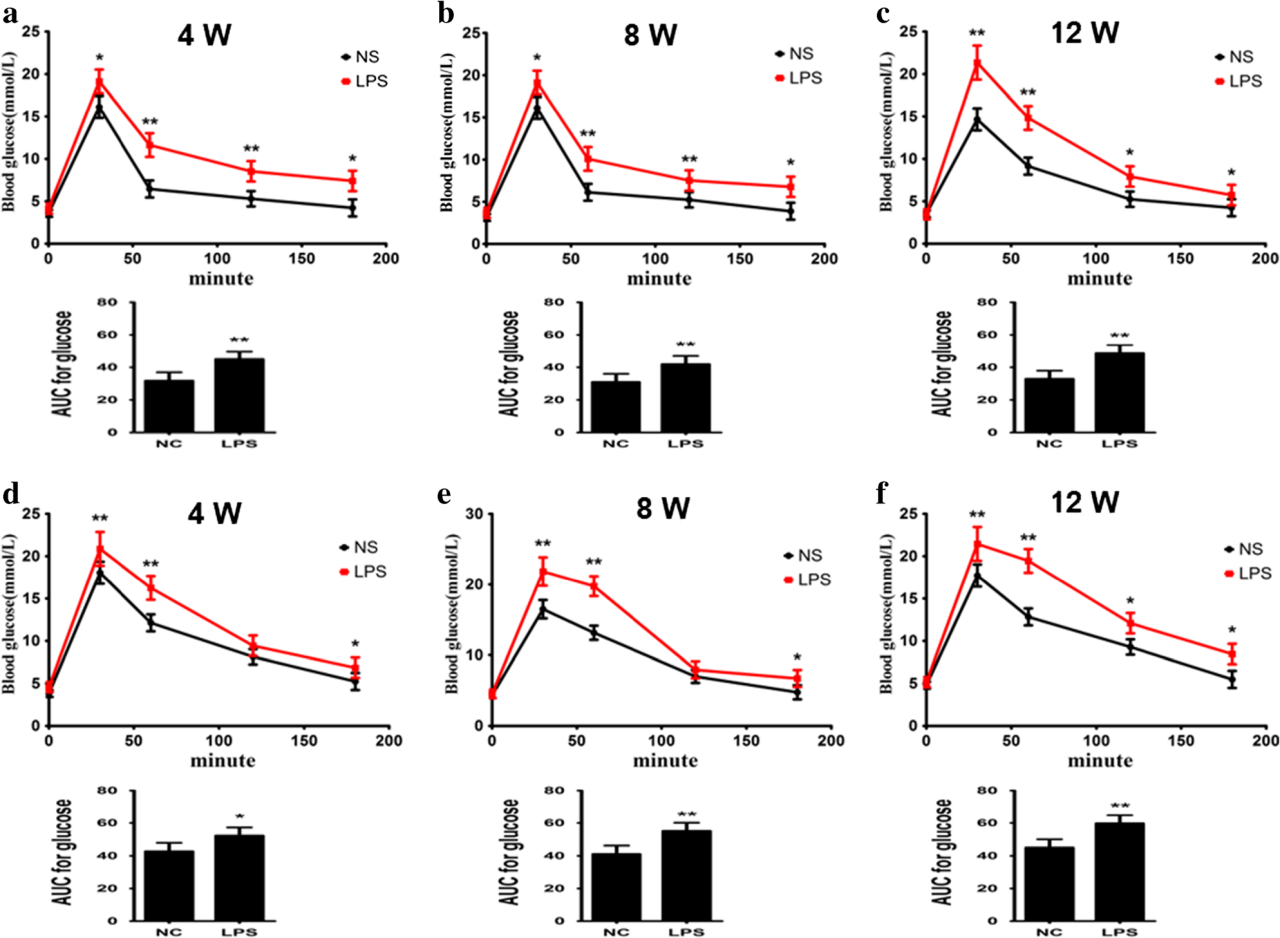
Maternal inflammation during pregnancy enhances offspring mouse blood glucose and glucose AUC. Glucose AUC levels and blood glucose levels results for 4-week (**a**), 8-week (**b**), and 12-week-old (**c**) female offspring mice and those of males 4 weeks (**d**), 8 weeks (**e**), and 12 weeks old (**f**). *p < 0.05, **p < 0.01*, compared with the control group (x ± s, n = 16).*

Glucose metabolism tests were measured to demonstrate obesity-induced glucose metabolism disorder [33, 34]. The fasting blood glucose of the all-of-weeks-age mice in the LPS group had modicum increased, but there was no significant difference. During the oral glucose tolerance test (OGTT), glucose levels peaked at 30 min and then gradually returned to baseline by 180 min in the two groups of all of weeks-old offspring mice (Fig. 4a–f). Blood glucose concentration in the 30 to 180 min and blood glucose response area under the curve (AUC) had increased significantly in all of weeks-old offspring mice; male mouse blood glucose in 120 min in the LPS group of 8-week-old and that of females 12 weeks old have been eliminated (*p <* 0.05) (Fig. 4a–f). Our results suggest that LPS stimulation during pregnancy led to abnormal glucose in the offspring mice and reduced glucose tolerance.

### RT-PCR and Western Blot Analysis of the Expression Quantity of PTX3

**Fig. 5:**
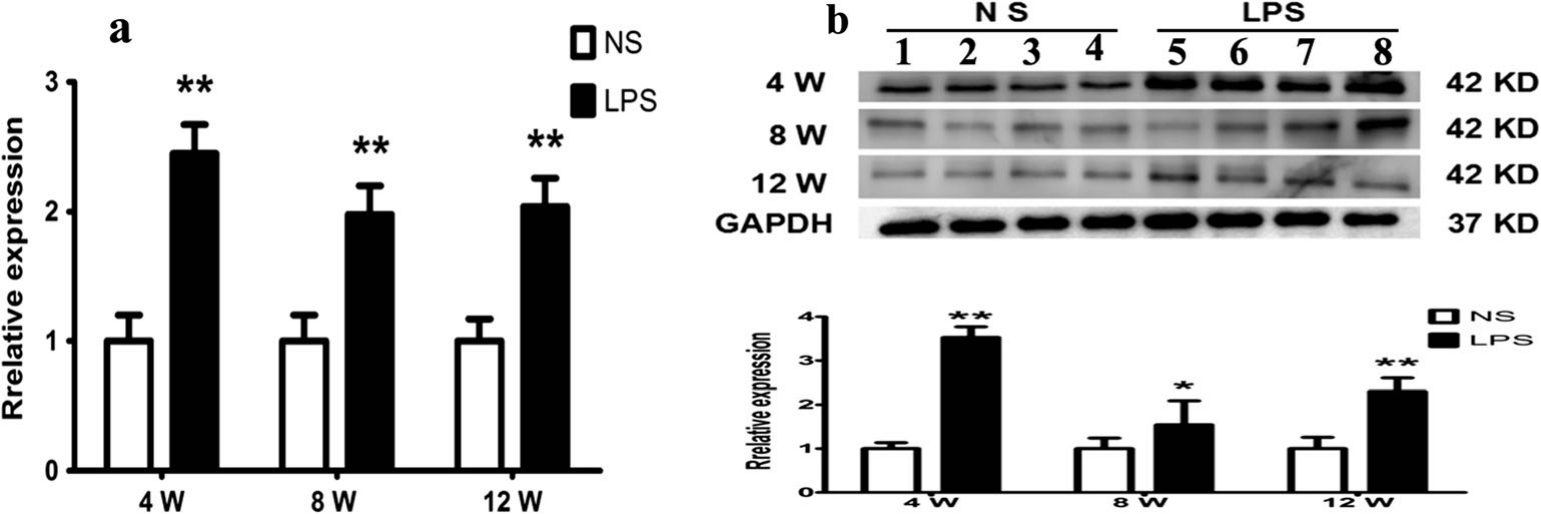
Maternal inflammation during pregnancy enhances the expression of PTX3 of adipose tissue in offspring mouse. The protein expressions of PTX3 in the offspring male epidydimal adipose tissue were assessed by immunoblotting and RT-PCR. Relative expression levels of the PTX3 mRNA in the epidydimal adipose tissue of the male offspring (**a**). Relative expression levels of PTX3 protein in the epidydimal adipose tissue (**b**).*p < 0.05, **p < 0.01*, compared with the control group (x ± s, n = 16).*

Furthermore, previous studies have demonstrated that inflammatory factor PTX3 plays an important role in both inflammation and obesity by previously reported literature [34]. Correlation analysis showed that LPS could induce PTX3 expression in macrophage cells by activating the transcription factor NF-κB induction [35]. Our results show that the levels of mRNA and protein expression of PTX3 in the adipose tissue were significantly increased in the offspring mice of the LPS group at all weeks of age (*p <* 0.05) (Fig. 5a, b). Our findings were consistent with previously reported literature.

### RT-PCR and Western Blot Analysis of Adipocyte Differentiation Markers

**Fig. 6:**
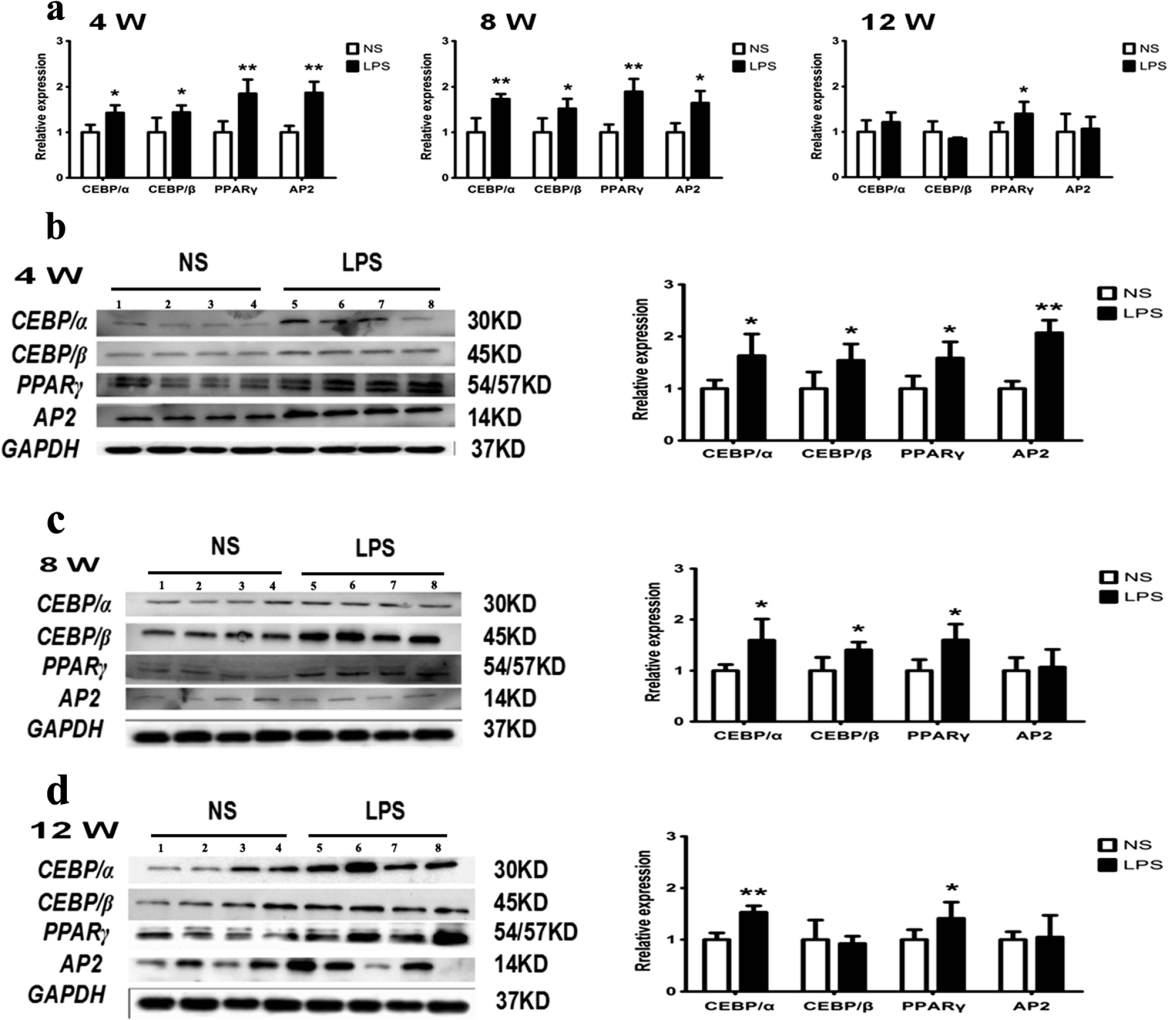
Adipocyte differentiation markers are abnormally increased in adipose tissue of mouse offspring. Relative expression levels of the adipose differentiation marker mRNA in the all-weeks male offspring (**a**). Relative expression levels of the adipose differentiation marker protein in the all-weeks male offspring (**b** 4 weeks; **c** 8 weeks; **d** 12 weeks).*p < 0.05, **p < 0.01*, compared with the control group (x ± s, n = 12).*

The incidence of obesity is directly related to the expression level of adipose differentiation markers [36]. Adipose differentiation markers which are overexpressed will be more likely to transform preadipocytes into adipocytes that aggravate the incidence of obesity. The levels of mRNA expression of CEBP/α, CEBP/β, PPARγ, and AP2 in the adipose tissue were significantly increased in the offspring of the LPS group at 4 and 8 weeks of age and the expression of PPARγ at 12 weeks of age (*p <* 0.05) (Fig. 6a). The levels of protein expression of CEBP/α, CEBP/β, PPARγ, and AP2 in the adipose tissue were significantly increased in the offspring of the LPS group at 4 weeks of age and the expression of CEBP/α, CEBP/β, and PPARγ in 8 weeks of age (*p <* 0.05) (Fig. 6b, c). The levels of protein expression of CEBP/β, AP2, and PPARγ in the adipose tissue were significantly increased in the offspring of the LPS group at 12 weeks of age (*p <* 0.05) (Fig. 6d). All the above results demonstrated that the offspring of LPS-treated mothers will cause an obvious offspring mouse obesity and metabolic disorders.

### RT-PCR and Western Blot Analysis of PTX3 and Adipocyte Differentiation Markers in the 3T3-L1 Cells That Were Transfected with siRNA and PTX3 Overexpression Plasmid

**Fig. 7:**
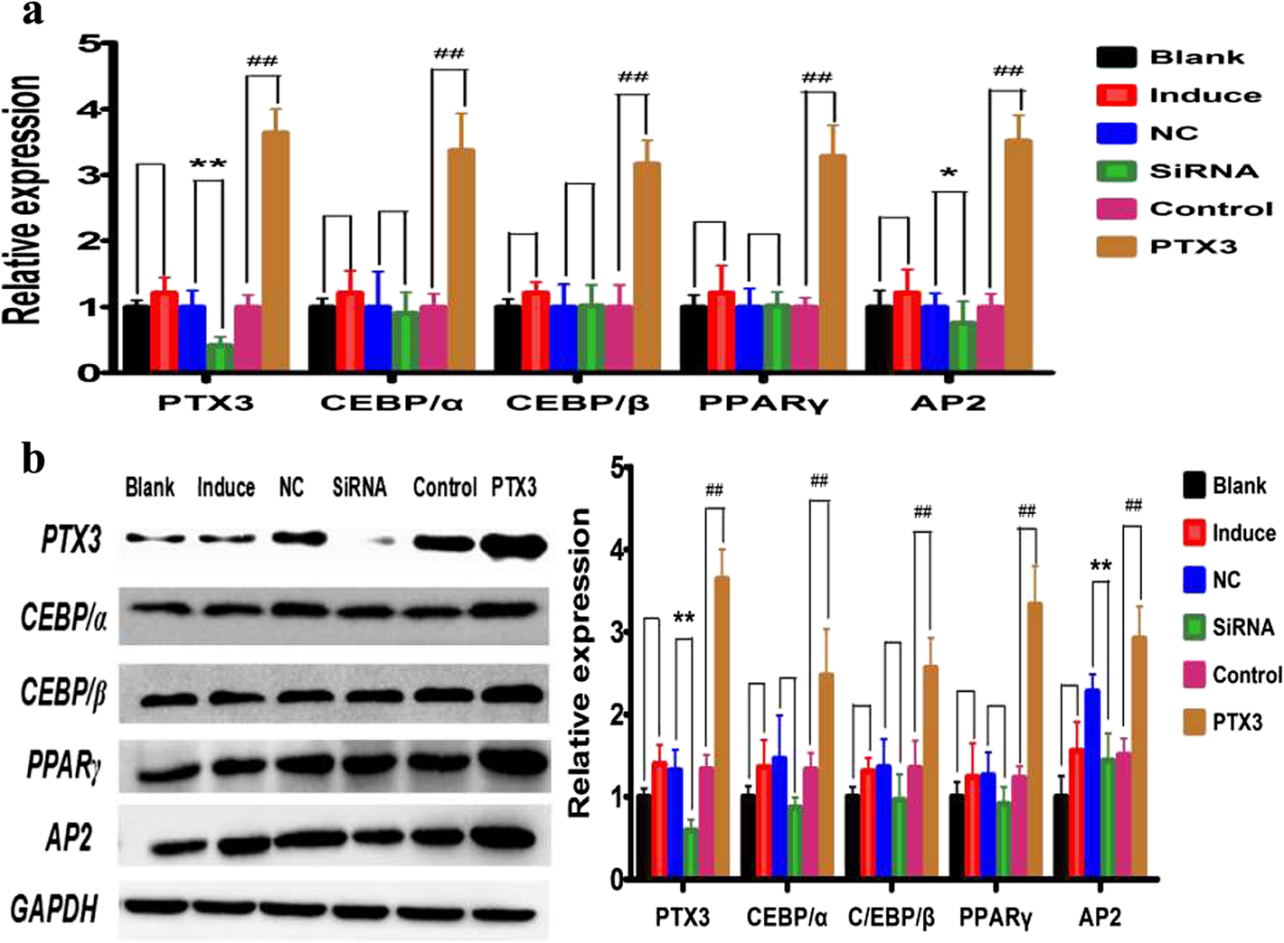
PTX3 activates the expression of adipocyte differentiation markers in 3T3-L1. Our previous experimental result had found that offspring of LPS-treated mothers exhibited moderate increase in the expression of both adipocyte differentiation markers and PTX3 of male epididymal fat tissue, so we speculate that a close relationship between PTX3 and adipocyte differentiation markers was existent. To test the hypothesis, the expression of adipose differentiation markers (AP2, PPARγ, CEBP/α, and CEBP/β) that was disposed with transfection of siRNA and PTX3 overexpression plasmid was measured in 3T3-L1 cells by western blotting and PT-PCR, the mRNA levels (**a**) and protein levels (**b**) in 3T3-L1 cells. *Blank*, normal medium; *induced*, induction reagent; *NC*, negative control, *siRNA*, transfection siRNA; *control*, PTX3 empty vector, *PTX3*, PTX3 overexpression plasmid. *p < 0.05, **p < 0.01*, compared with the NC group (x ± s, n = 5),* ^*#*^ *p < 0.05,* ^*##*^ *p < 0.01, compared with the control group (x ± s, n = 5).*

We know that PTX3 plays an important role in inflammatory response [37, 38]. PTX3 activates and regulates the complement cascade by interacting with C1q and Factor H to produce the ability of immune regulation [17]. Adipose differentiation markers causing changes in fat metabolism are mainly through the interaction regulation between multiple proteins [39]. However, the activity of PTX3 and the specific mechanisms in that intrauterine inflammation causes offspring mouse obesity remain unknown. Through the experimental data, it is easy to hypothesize whether PTX3 and adipocyte differentiation markers exist in a close relationship because of the simultaneous increase. The expression levels of PTX3, CEBP/α, and AP2 mRNA and protein were significantly decreased in the 3T3-L1 cells in the siRNA group (*p <* 0.05) (Fig. 7a, b). The expression levels of PTX3, AP2, PPARγ, CEBP/α, and CEBP/β mRNA and protein were significantly increased in the 3T3-L1 cells that were in the PTX3 overexpression plasmid group (*p <* 0.05) (Fig. 7a, b). It is well-known for us that overexpression of fat differentiation markers will cause obesity; there is no denying that PTX3 directly regulates the expression of fat differentiation markers, so our findings indicated that gestation inflammatory exposure may predispose offspring to obesity when inflammatory factor PTX3 regulates the upregulation expression of adipogenic differentiation markers. In other words, PTX3 can increase the susceptibility to obesity by regulating the expression of adipogenic markers.

### Effects of siRNA and PTX3 Plasmid Transfection on Cell Proliferation, Apoptosis, and Differentiation in 3T3-L1 Cells

**Fig. 8:**
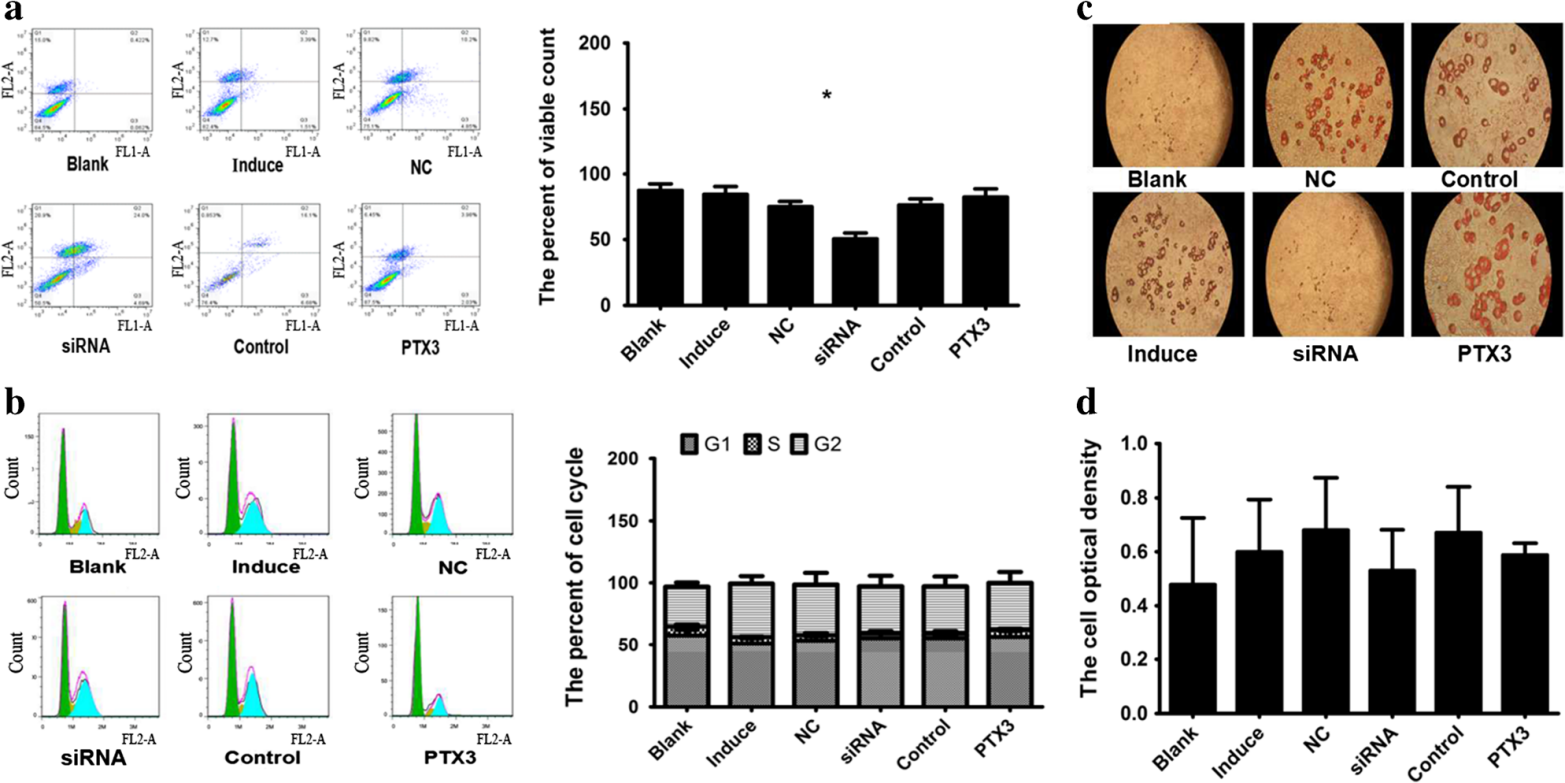
siRNA and PTX3 plasmid transfection did not affect cell apoptosis and proliferation in 3T3-L1 cells. siRNA transfection induces cell apoptosis, and PTX3 plasmid transfection promoted cell proliferation in 3T3-L1 cells. The expression level of PTX3 and adipose differentiation marker synchronization increased, whether it is for this reason that it is through the influence on the function of the process of cell development including cell proliferation, differentiation, apoptosis. So we measured the impact of PTX3 overexpression and lower expression on cell apoptosis (**a**), the cell cycle (**b**), cell differentiation (**c**) and cell proliferation (×400) (**d**) to ulteriorly define the relationship between markers of adipose differentiation. *Blank*, normal medium; *induced*, induction reagent; *NC*, negative control; *siRNA*, transfection siRNA, *control*, PTX3 empty vector; *PTX3*, PTX3 overexpression plasmid.*p < 0.05, **p < 0.01*, compared with the NC group (x ± s, n = 5).*

The effect of PTX3 expression on cell proliferation, apoptosis, and differentiation in 3T3-L1 cells is expected to be validated as to whether or not PTX3 adjusts the expression of fat differentiation markers by affecting the growth of cells. Compared with the NS group, transfection with siRNA had significant promoted cell apoptosis and inhibited cell differentiation (*p <* 0.05) **(**Fig. 8a, c), but with no significant effect on the cell cycle and proliferation of 3T3-L1 cells (*p* > 0.05) (Fig. 8b, d). Compared with the control group, transfection with the PTX3 plasmid had no significant effect on apoptosis, cell cycle, and proliferation of the 3T3-L1 cells (*p <* 0.05) (Fig. 8a–d), but it significantly inhibited cell differentiation (*p <* 0.05) (Fig. 8c). The results showed that the regulation of PTX3 on adipose differentiation markers was not related to regulate the growth status of cells and mainly through the impact of cell differentiation to achieve.

### RT-PCR and Western Blot Analysis of PTX3 and Western Blot Analysis of the MAPK Pathway (p38, JNK, ERK1/2)

**Fig. 9:**
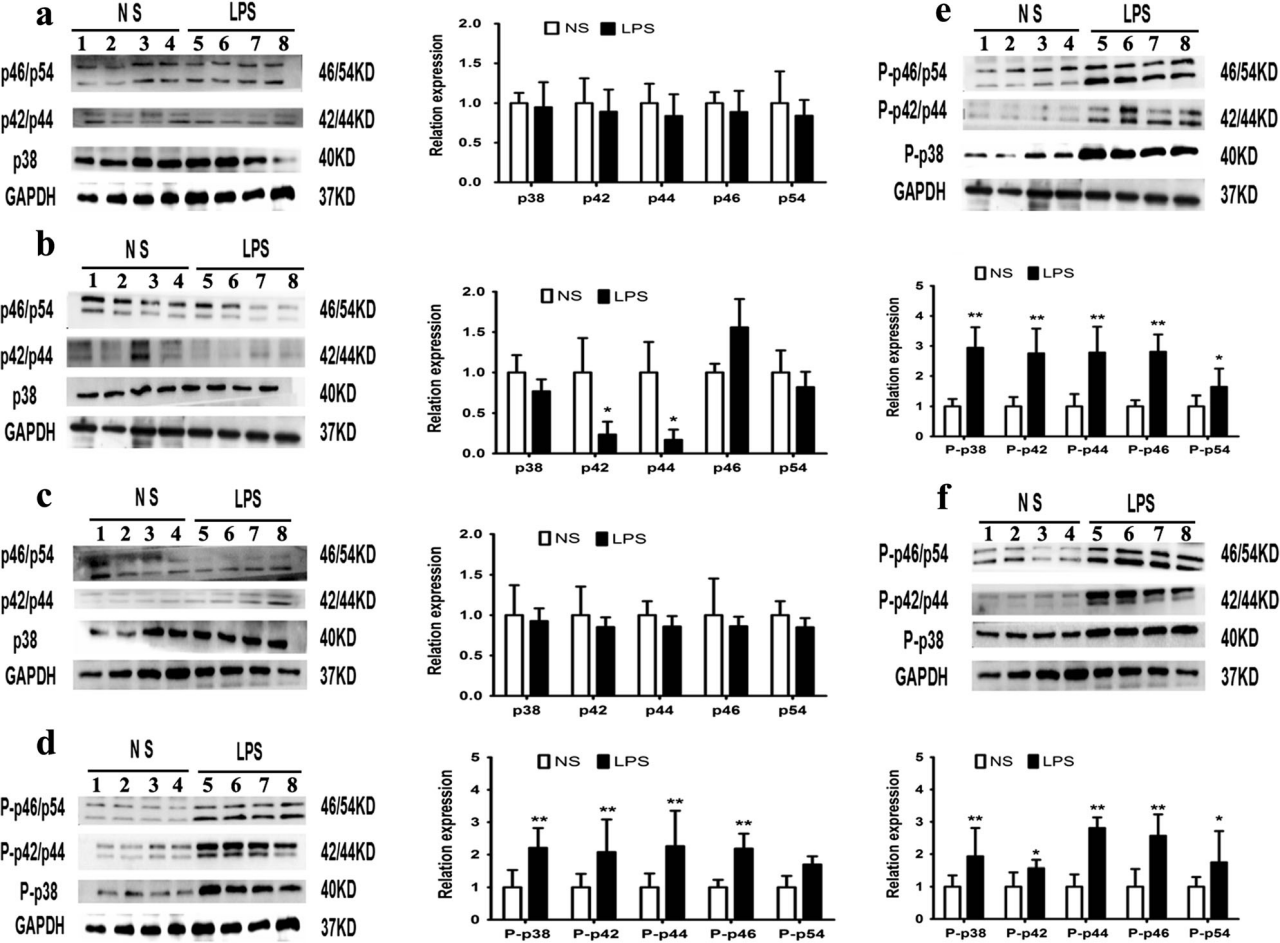
The phosphorylation of mitogen-activated protein kinase (MAPK) pathway proteins (p38, JNK/SAPK, and ERK1/2) was significantly increased in the offspring of the LPS group at every age examined. Expression of non-phosphorylation MAPK pathway proteins in the male epidydimal adipose tissue of male offspring at 4 weeks (**a**), 8 weeks (**b**), and 12 weeks (**c**). Relative expression of phosphorylated MAPK pathway protein at 4 weeks (**d**), 8 weeks (**e**), and 12 weeks (**f**).*p < 0.05, **p < 0.01*, compared with the control group (x ± s, n = 16).*

We know that the expression of PTX3 in the inflammatory response is mainly controlled through the MAPK pathway [40, 41]. Our results demonstrate that the protein expression of non-phosphorylated in the adipose tissue was relatively less in the offspring of the LPS group at all weeks of age (*p <* 0.05) (Fig. 9a–c). The protein expression of non-phosphorylated ERK1/2(p42/p44) in the adipose tissue was significantly decreased in the offspring of the LPS group at 8 weeks of age (*p <* 0.05) (Fig. 9c). The protein expression of phosphorylated p38, ERK1/2(p42/p44), and JNK/SNPK (p46/p54) in the adipose tissue was significantly increased in the offspring of the LPS group at all weeks of age (*p <* 0.05) (Fig. 9d–f). It can be concluded that the overexpression of PTX3 is most likely regulated by the MAPK pathway in inflammatory obesity. Certainly, more experiments are expected.

## DISCUSSION

The relationship between obesity and inflammation requires an in-depth study. Obesity as a chronic low-level inflammatory disease has become a recognized fact [42], but whether inflammation directly leads to obesity has rarely been reported. A large amount of experimental proof has suggested that the occurrence of many adult chronic diseases is closely related to the growth environment of the fetus *in utero*. Intrauterine inflammation causes metabolic diseases and obesity in offspring and is an essential independent factor in congenital hereditary obesity [43–45]. Adipose tissue is a key regulator of energy stability; it can not only save energy *via* metabolism but also produce a variety of cytokines that are involved in systemic metabolic activity and inflammatory responses, and inflammation can further aggravate the extent of obesity [46–48], thus forming a vicious cycle. In this study, the level of PTX3 was significantly increased in the adipose tissue of obese offspring mice, and its inflammatory reaction was confirmed in the adipose tissue. The body weight, visceral fat wet weight, fat coefficient, and blood lipid levels were abnormally increased in the LPS group offspring mice. Additionally, the offspring mice displayed impaired glucose tolerance, increased volume of adipocytes, and poor glucose metabolism (Figs. 1, 2, 3, and 4).

In a previous study using an obesity-induced inflammation model, overexpression of PTX3 regulated the cell uptake of OX-LDL to prevent the outflow of cholesterol through activation of the ERK1/2 pathway [49]. In patients who are obese and who have diabetes, tumor necrosis factor-α (TNF-α) can induce an increase in PTX3 in smooth muscle cells (HASMC) through the MAPK pathway in response to inflammation [50]. In obesity-induced atherosclerosis, high-density lipoprotein (HDL) is able to promote high expression levels of PTX3 by activating the PI3K/AKT (phosphatidylinositol-3-kinase/protein kinase-B) pathway, leading to an increase in the expression of APO-1 *in vivo* [51]. All of these studies indicate that PTX3 plays an important role in the development and progression of obesity. The present study confirmed that the expression of PTX3 in the offspring mice was significantly increased (Fig. 5a, b).

Studies have shown that blocking the expression of fatty acid-binding protein (FABP4/AP2) could improve inflammation-induced obesity by modulating the upregulated expression of uncoupling protein 2 (UCP2) [52]. Peroxisome proliferator-activated receptor γ (PPARγ) is the main regulator of fat metabolism and differentiation, and preadipocytes will be more likely to differentiate into mature adipocytes when the expression of PPARγ is increased [53, 54]. The CCAT enhancer-binding (C/EBP) family plays an important role in regulating the differentiation and formation of adipocytes [55]. The mutual regulation and influence of the adipocyte differentiation markers (AP2, PPARγ, CEBP/α, and CEBP/β) regulate preadipocyte differentiation into mature adipocytes [54]. Therefore, PTX3 may target the expression of adipocyte differentiation markers to regulate the development of obesity. In this experiment, we found that the expression levels of PPARγ, CEBP/α, and CEBP/β in 4-week-old mice and of PPARγ and CEBP/α in 8-week-old mice in the LPS group were significantly higher than the NS group (Fig. 6a–d). Plasmid overexpression experiments have shown that PTX3 has a positive regulatory effect on adipogenic markers (Fig. 7a, b) and does not significantly impact adipocyte proliferation, the cell cycle, and apoptosis (Fig. 8a–d, f). However, it significantly promoted the differentiation of adipocytes (Fig. 8e). We also found that low expression of PTX3 had no effect on cell cycle and proliferation (Fig. 7c–d, f). However, low expression of PTX3 could alter the expression of adipogenic markers (AP2) and promote adipocyte apoptosis to significantly decrease the degree of preadipocyte differentiation into mature adipocytes in siRNA interference experiments. Therefore, we concluded that PTX3 could promote the progression of obesity by regulating the expression levels of adipocyte differentiation markers (AP2, PPARγ, CEBP/α, and CEBP/β).

PTX3 in the inflammatory response is mainly controlled through the MAPK pathway [40, 41]. Therefore, we measured MAPK pathway-related protein levels in 4-, 8-, and 12-week old mouse adipose tissue of the LPS group. The results showed that the phosphorylation of mitogen-activated protein kinase (MAPK) pathway proteins (p38, JNK, and ERK1/2) was significantly increased in the offspring of the LPS group at every age examined **(**Fig. 9a–f). Therefore, it can be concluded that the overexpression of PTX3 is most likely regulated by the MAPK pathway in inflammatory obesity.

In conclusion, prenatal exposure to LPS results in PTX3 activation in the adipose tissues of the offspring mice. High expression of PTX3 promotes the formation of adipocytes through the regulation of differentiation marker expression, and it eventually leads to obesity. This effect of PTX3 is most likely regulated by the MAPK pathway. Therefore, PTX3 plays an important role in increasing obesity susceptibility. This study has shown that prenatal exposure to LPS will lead to obesity and that PTX3 plays an important regulatory role in obesity. Moreover, this study has elucidated the mechanism of action and provided new targets for obesity drug therapy. This study has also generated the theoretical basis for early clinical anti-inflammatory intervention in pregnancy, providing a new way of thinking and a new direction to treatment of obesity.

In this article we discuss whether inflammation will directly lead to obesity, Our study confirmed that PTX3 is most likely caused by MAPK pathway hyperactivation can increase the susceptibility to obesity by regulating the expression of adipogenic markers.

## Abbreviations

English abbreviation: English full name
AP2: Adaptin 2
CEBP/α: CCAAT/enhancer-binding protein α
CEBP/β: CCAAT/enhancer-binding protein β
AUC: Area under the curve
BSA: Bovine serum albumin
HDL: High-density lipoprotein
HE: Hematoxylin-eosin staining
SDS: Sodium dodecyl sulfate
LPS: Lipopolysaccharide
MTT: MTT assay
NS: Normal saline
PBS: Phosphate-buffered saline
PPARγ: Peroxisome proliferator-activated receptor γ
RT-PCR: Real-time polymerase chain reaction
TC: Total cholesterol
TG: Triglyceride
PTX3: Pentraxin-3
MyD88: Myeloid differentiation factor 88
TLR9: Toll-like receptor 9
IL-12: Interleukin-12
IRF3: Interferon regulatory factor 3
TNF-α: Tumor necrosis factor α
LPS: Lipopolysaccharedes
eWAT: Epididymal white adipose tissue
DMEM: Dulbecco’s modified eagle medium
PI: Propidium iodide
PMSF: Phenylmethanesulfonyl fluoride
IBMX: Isobutylmethylxanthine
DEX: Dexamethasone
INS: Insulin
MAPK: Mitogen-activated protein kinase
JNK/SAPK(p42/p44): C-Jun N-terminal kinase/stress-activated protein kinase
ERK1/2(p46/p54): Extracellular signal-regulated kinase 1/2
OX-LDL: Oxidized low-density lipoprotein
HASMC: Human aortic smooth muscle cells
UCP2: Uncoupling protein 2
NF-κB: Nuclear factor kappa
siRNA: Short interfering RNA
